# Functional atlases for analysis of motor and neuropsychological outcomes after medial globus pallidus and subthalamic stimulation

**DOI:** 10.1371/journal.pone.0200262

**Published:** 2018-07-13

**Authors:** Claire Haegelen, Clément Baumgarten, Jean-François Houvenaghel, Yulong Zhao, Julie Péron, Sophie Drapier, Pierre Jannin, Xavier Morandi

**Affiliations:** 1 Department of Neurosurgery, CHU Pontchaillou, Rennes, France; 2 INSERM, LTSI U1099, Faculté de Médecine, Rennes, France; 3 University of Rennes I, Rennes, France; 4 Department of Neurology, CHU Pontchaillou, Rennes, France; 5 Behavior and Basal Ganglia host team 4712, University of Rennes 1, Rennes, France; 6 Swiss Centre for Affective Sciences, Geneva, Switzerland; Hospital General Dr. Manuel Gea Gonzalez, MEXICO

## Abstract

Anatomical atlases have been developed to improve the targeting of basal ganglia in deep brain stimulation. However, the sole anatomy cannot predict the functional outcome of this surgery. Deep brain stimulation is often a compromise between several functional outcomes: motor, fluency and neuropsychological outcomes in particular. In this study, we have developed anatomo-clinical atlases for the targeting of subthalamic and medial globus pallidus deep brain stimulation. The activated electrode coordinates of 42 patients implanted in the subthalamic nucleus and 29 patients in the medial globus pallidus were studied. The atlas was built using the representation of the volume of tissue theoretically activated by the stimulation. The UPDRS score was used to represent the motor outcome. The Stroop test was represented as well as semantic and phonemic fluencies. For the subthalamic nucleus, best motor outcomes were obtained when the supero-lateral part of the nucleus was stimulated whereas the semantic fluency was impaired in this same region. For the medial globus pallidus, best outcomes were obtained when the postero ventral part of the nucleus was stimulated whereas the phonemic fluency was impaired in this same region. There was no significant neuropsychological impairment. We have proposed new anatomo-clinical atlases to visualize the motor and neuropsychological consequences at 6 months of subthalamic nucleus and pallidal stimulation in patients with Parkinson's disease.

## Introduction

High frequency deep brain stimulation (DBS) is an effective treatment for patients with severe disabling movement disorders refractory to medical treatment. For 25 years, subthalamic nucleus (STN) stimulation has proved its efficacy on the triad of Parkinson's symptoms [1,2] notwithstanding its potential neuropsychological and psychiatric secondary effects [3–8]. Patients with severe disabling Parkinson’s disease (PD) and severe cognitive impairment and/or axial symptoms could be operated on medial globus pallidus (GPm) when STN DBS was contraindicated [9]. Even if many authors [10–12] have reported little deterioration after GPm DBS, it seems important to analyze more accurately the effect of stimulation on motor and neuropsychological aspects in different GPm areas using 3D functional atlases. Functional atlases to analyse motor outcomes after STN stimulation exist, but no functional atlas has been built, to our knowledge, about GPm DBS.

Functional atlases introduced by Nowinski et al. [13–15] integrate information such as position of electrode contacts, stimulation or electrophysiological recordings to determine the efficacy zones of DBS. Functional atlases have been developed in a majority of cases for STN DBS because of the small size of this nucleus and the difficulty of its direct targeting based on MRI contrast, and for better understanding of the secondary neuropsychological side effects of the stimulation. We previously described a methodology for the creation of functional atlases in STN DBS which correlate clinical scores with active electrode contacts, in order better to understand clinical effects of stimulations according to their specific locations. In particular, we showed that targeting the postero-superior region of the STN induced discrepancy between a very good motor improvement and a deterioration of the semantic and phonemic fluencies in the same region [16]. Inversely, stimulating the ventral part of the STN resulted in better postoperative fluencies results but less motor improvement. Conversely, Mikos et al. [17] demonstrated a negative correlation between the volume of stimulation in the ventral STN and letter fluency changes, whereas they observed better fluency performance when increasing the stimulation volume at the optimal STN zone. Neither in Mikos et al. [17] nor Okun et al. [9], have the neuropsychological changes been correlated with the motor outcome.

Thus, the objective of the study was to create functional atlases, accounting not only for electrode location, but also the electrical distribution of the electrode, represented by the volume theoretically activated by each stimulation. This new representation of motor and neuropsychological effects is aimed at helping the clinician to understand and choose a difficult compromise between therapeutic and side effects as explained above, in the particularly deep and small area of the basal ganglia.

## Materials and methods

### Patients

Twenty-nine PD patients who underwent GPm DBS and 42 patients, who underwent STN DBS at the Rennes University Hospital between 2006 and 2015, were enrolled in the study. Two institutional review boards approved the study, the “Comité de Protection des Personnes Ouest V” from Rennes’ Hospital, and the “Agence Française de Sécurité Sanitaire des Produits de Santé” (reference B110464-80). All patients met the criteria of the United Kingdom Parkinson’s Disease Society brain bank for idiopathic PD [18]. For the GPm-DBS group, the female-to-male ratio was 17–12 with a mean age of 60.5 ±7.2 years and mean disease duration of 12.8 ±6.2 years at the date of the surgery. STN DBS was contraindicated for all patients of this group, due to cognitive impairment (Mattis dementia rating scale -MDRS- ≤ 130 and/or an impairment of at least 50% of executive functions assessing) and/or dopa-resistant axial motor symptoms (dysarthria, freezing, falls) at baseline [19]. For the STN-DBS group, the female-to-male ratio was 17–25 with a mean age of 55.6 ±7.6 years and mean disease duration of 10.1 ±3.8 years at the date of the surgery. After a complete description of the study, written informed consent was obtained from each participant and the study was conducted in accordance with the Declaration of Helsinki. Clinical assessment was performed before (mean 5 ±4.7 months) and after surgery (mean 6 ±2 months) and was conducted in accordance with the Core Assessment Program for Intracerebral Transplantation (CAPIT) [20] (Table 1).

**Table 1:** Clinical data concerning 29 patients with GPm DBS and 42 patients with STN DBS.

Target	^a^Preop ^b^STN	^c^Postop ^b^STN	^a^Preop ^d^GPm	^c^Postop ^d^GPm
**Age at surgery** **(years ±** ^e^**SD)**	55.6 ±7.6		60.5 ±7.2	
**Disease Duration** **(years ±** ^e^**SD)**	10.1 ±3.8		12.8 ±6.2	
^f^**UPDRS III**	31.4 ±11.6	16 ±9.7 *	42.1 ±17.5	28.7 ±11.6 *
^g^**MDRS**	140.5 ±2.8	141 ±3.4	131.6 ±7.5	132.6 ±6.9
**Stroop test**	0.8 ±7	1 ±6.1	-3.4 ±9.1	-3.9 ±8
**Semantic Fluency**	31.9 ±10.3	29.5 ±9.3	23.4 ±10.2	21.8 ±8.8
**Phonemic Fluency**	20.3 ±6.6	20.7 ±6.4	14.4 ±6.8	12.5 ±5.8
**Mean levodopaequivalent daily (**^h^**mg)**	1244.2 ±617.5	798.4 ±472.9	1450.1 ±654.3	1395.4 ±449.4

Values are mean ±interquartile range.

* means a statistical significant change after DBS (p<0.05)

^a^ Preop = preoperative

^b^ STN = subthalamic nucleus

^c^ Postop = postoperative

^d^ GPm = medial globus pallidus

^e^ SD = standard deviation

^f^ UPDRS III = Unified Parkinson’s Disease Rating Scale Part III

^g^ MDRS = Mattis Dementia Rating Scale

^h^mg = milligrammes

To build the functional atlases, we included the Unified Parkinson’s Disease Rating Scale part III (UPDRS III) [21], tests assessing executive functions, including phonemic (letter *p*) and semantic (animals) verbal fluency tasks (2-min version) [22] and Golden’s version of the Stroop Interference Test [23]. We built the functional atlases on similar cognitive tests frequently described in Parkinson’s disease and after DBS [24, 25]. For the UPDRS III score, patients were tested by a neurologist before surgery without medication (Dopa Off) and at 6 months after surgery with stimulation (DBS On) and without medication. For each neuropsychological score, patients were tested with medication (Dopa On) prior to surgery and after it under stimulation (DBS On), also with medication. The motor and neuropsychological testing were done the same week pre- and postoperatively.

In the STN group, the mean levodopa equivalent daily dose was 1244.2 ±617.5 mg per day preoperatively, and 798.4 ±472.9 mg per day postoperatively, i.e. a decrease of 35.8%. In the GPm group, the mean levodopa equivalent daily dose was 1450.1 ±696.8 mg per day preoperatively, and 1395.4 ±431.9 mg per day postoperatively, i.e. a decrease of 3.7%.

### Surgical procedure

The surgical procedure consisted first in attaching a stereotactic Leksell frame to the patient’s head under local anaesthesia, then implanting bilateral quadripolar DBS electrodes (3387 Medtronic for the GPm and 3389 Medtronic for the STN, Minneapolis, MN, USA) in the postero-ventral part of the two GPm or in the postero-lateral part of the two STN in a single operating session [1,26]. Anti-parkinsonian treatment was stopped the evening before surgery. The patient was awake throughout the procedure; the effect of stimulation on rigidity was assessed by passive movement of the controlateral wrist. Programmable pulse generators (Medtronic) were implanted in the subclavicular region and connected to the electrodes.

#### Imaging data and electrode contact localization

All patients had one preoperative 3-T T1-weighted MR (1 mm x 1 mm x 1 mm, Philips Medical Systems) and two CT scans (0.5 mm x 0.5 mm x 0.6 mm in preoperative acquisition and 0.44 mm x 0.44 mm x 0.6 mm in postoperative acquisition, GE Healthcare VCT 64). Preoperative scans were acquired after attachment to the patient's head of a stereotactic Leksell frame, under local anaesthesia. All images were de-noised using the non-local means algorithm [27] and a bias correction algorithm based on intensity values [28] was also applied to MR images.

We assembled information acquired from a population of patients onto one spatially normalized reference image (i.e. template). As a common space, we chose a multi-subject MR template created from a population of patients with PD (named the ParkMedAtlis template), with the segmentation of the basal ganglia and deep brain structures validated by a previous study [29]. After a preliminary step of automatic electrode contact segmentation based on image processing operations, combinations of linear and non-linear registrations allowed each stimulated contact to be warped in the ParkMedAtlis template [30]. Both the contact segmentation and the registration algorithms had already been meticulously and successfully validated [16]. Briefly, the registration workflow was composed of a linear CT to MRI registration, a global affine MR-to-template transformation, a local affine MR-to-template transformation with a mask on deep structures, and a final non-linear registration. Using this procedure, the contact positions could be precisely warped into the ParkMedAtlis template, taking the anterior commissure (AC) as the origin. The distance between the AC and the posterior commissure (PC) of the ParkMedAtlis template was 26 millimeters.

#### Modeling of stimulation

Several methods have been proposed for modeling the electric field during DBS [31–33]. To model the neural response, the activating function was used in Butson et al. [34], who proposed modeling based on a finite element model. At our site, mono-polar stimulation is generally proposed. Given the conclusion of Butson et al. [34] on the complexity of correctly simulating the stimulation phenomena, we decided to model the electric field using a single pre-defined 3D Gaussian. The parameters of the electrodes were: for the GPm, Medtronic® 3387 (4 contacts per electrode, 1.27 mm diameter, 1.5mm length, 1.5 mm between the contacts) and for the STN, Medtronic® 3389 (4 contacts per electrode, 1.27 mm diameter, 1.5mm length, 0.5 mm between the contact), the two being used with a monopolar stimulation protocol. Based on these parameters and on the quasi-static potential equation, the stimulation influence covers approximately a 3 mm-radius sphere around each stimulation contact.

#### Functional atlases

To build the functional atlases, a relative score of improvement or deterioration *S*% was defined for each score *S*. Given that the patients were not all responding the same way to treatment, an average of both scores was necessary as follow:

- for the UPDRS III:
  $$S\%=-(\frac{S_{dopa-off-DBS-on}^{post}-S_{dopa-off}^{pre}}{S_{\max}})$$
- for the other scores:
  $$S\%=\frac{S_{score}^{post}-S_{score}^{pre}}{S_{\max}{}_{}}$$
  where $S_{dopa-off-DBS-on}^{post}$ and $S_{dopa-off}^{pre}$ were the values of the postoperative and preoperative scores without medication respectively, $S_{score}^{post}$ and $S_{score}^{pre}$ were the values of the postoperative and preoperative neuropsychological scores respectively, *S*_max_ was the maximum of the clinical test. For example, the maximum of the UPDRS III was 70. For the Stroop test, we chose arbitrary minimum and maximum values, i.e. -20 and 20 respectively. For the semantic and phonemic fluencies, the minimum and maximum values were chosen at 0 and 30 respectively.

Our aim is to create general atlases easy for clinicians to read and interpret. The functional atlases should take into account the number of patients, the stimulation zone for each patient, as well as all responses to clinical testing of each patient. We obtained two atlases per score, the first one (the 'Functional Atlas') representing the postoperative change of the score per voxel, and the second one (the 'Patient Atlas') representing the number of patients per voxel used for the final score computation. To calculate the value of each voxel in each ‘Functional Atlas’, (i) we determine for each patient the voxels activated by the electrical stimulation (ii) if the voxel was activated by several patients, we calculated the average of the values. The value at a given voxel does not represent a probability but a global efficacy score. We also proposed a second atlas, the ‘Patient Atlas’ representing only the number of patients per voxel, in other words the region with a maximum of patients for a given target. The two atlases are 3D maps in which it is possible to navigate to find the region with the maximum of patients in the ‘Patient Atlas’ and then, the corresponding region in the ‘Functional atlas’ with the value of the *S%* score.

Patients who did not have the same version pre- and postoperatively for the semantic (“animal”) and phonemic fluencies (“P”) were excluded from the verbal fluency ‘Patient Atlas’ and ‘Functional Atlas’. Thus, 23 patients with GPm DBS and 33 patients with STN DBS were included in the building of the verbal fluency atlas.

#### Statistical analysis

Analysis was performed using the non-parametric Wilcoxon test to compare the STN and GPm DBS on change scores (UPDRS III, MDRS, Stroop test, semantic and phonemic fluencies). Analysis was performed using the SPSS software. The level of significance was defined as less than 0.05.

## Results

### Statistical analysis of the motor and neuropsychological scores

All results are detailed in Table 1. STN and GPm DBS led to a significant improvement in motor functions (*p*<0.05). The other scores did not change significantly after STN or GPm DBS. The postoperative mean levodopa equivalent daily dose significantly decreased after STN DBS (*p*<0.05) but not after GPm DBS.

### Stimulation contact and parameters

Given the size of the GPm, 12 patients were stimulated on two contacts, 9 patients on 0 and 1 and 3 patients on 1 and 2 bilaterally with monopolar stimulation. The 17 other patients had only one contact stimulated bilaterally, as did the 42 patients with STN DBS.

Six months after surgery, the mean position of the active electrode's contacts was calculated by first making an average of the coordinates in each patient with double stimulated contacts, and secondly, an average of all the coordinates in all the patients. The mean coordinates and the average stimulation parameters of the active contact of GPm and STN DBS are detailed in Table 2.

**Table 2:** Mean (± standard deviation) coordinates and stimulation parameters of 29 patients with GPm DBS and 42 patients with STN DBS at 6 months postoperatively.

			Mean Coordinates			Average Parameters	
		Lateral (^a^mm)	Anterior (^a^mm)	Vertical (^a^mm)	Voltage (^b^V)	Frequency (^c^Hz)	Amplitude (^d^μs)
^e^**STN**	**Left**	-14.5 ±1.5	-16.7 ±2.1	-1.4 ±1.55	2.3 ±0.6	132.4 ±9.4	60.7 ±4.7
	**Right**	14 ± 2.6	-15.9 ±2.8	-1 ±2.8	2.4 ±0.6	134.3±14.1	61.4 ±6.5
^f^**GPm**	**Left**	-24.7 ±1.8	-12.1 ±2.4	-0.9 ±3.6	2.8 ±0.4	132 ±9.4	79.3 ±21.5
	**Right**	23.6 ±2	-11.6 ±4.2	0.55 ±4.2	2.8 ±0.4	132 ±9.4	77.6 ±20.5

Values are mean ±interquartile range.

The origin of the mean coordinates was the anterior commissure. The coordinates of the anterior-posterior direction were negative behind and positive in front of the anterior commissure. The coordinates of the lateral direction were negative on the left, positive on the right. The coordinates of the caudo-dorsal direction were negative under the anterior–posterior commissure line.

^**a**^*mm* = millimeters

^**b**^*V* = volts

^**c**^*Hz* = Hertz

^**d**^*μs* = microseconds

^**e**^*STN* = subthalamic nucleus

^**f**^*GPm* = medial globus pallidus

### Functional atlases

For the ‘Functional Atlas’, the color scale was dark blue (deterioration) to dark red (improvement). The ‘Patient Atlas’ was also coded using a color scale from dark blue (one patient) to dark red (highest number of patients for a given score). For instance, a voxel on the ‘Functional Atlas’ representing a dark red point of the score would represent only one patient on the 'Patient Atlas” (dark blue point). Inversely, a dark blue voxel on the ‘Functional Atlas’ would represent the highest number of patients in the ‘Patient Atlas’ (dark blue voxel) and would be more representative of the patient group. We obtained the values of the *S%* scores for the different tests assessed and the number of patients per voxel in the ‘Patient Atlas’, which are detailed in Table 3.

**Table 3:** Details of *S%* scores and number of patients for ‘Functional’ and ‘Patient’ atlases.

Scores *S%*	Total number of patients	‘^a^Funct Atlas’: Median (^b^Min-^c^Max) of *S%* score	‘Patients Atlas’: maximum of patients at one voxel	‘Funct Atlas’: *S%* scores at the red points in the ‘Patient Atlas’
**^d^STN**				
^e^**UPDRS III**	42	0.22 (-0.11 to 0.63)	20	0.17 to 0.3
**Stroop**	42	0 (-0.39 to 0.35)	20	-0.02 to 0.06
**Semantic Fluency**	33	-0.06 (-0.53 to 0.37)	19	-0.17 to -0.05
**Phonemic Fluency**	33	0 (-0.27 to 0.5)	19	0.03 to 0.07
^f^**GPm**				
^e^**UPDRS III**	29	0.17 (-0.12 to 0.61)	16	0.12 to 0.26
**Stroop**	29	-0.01 (-0.28 to 0.55)	15	0.005 to 0.03
**Semantic Fluency**	23	0 (-0.43 to 0.23)	13	-0.02 to 0.06
**Phonemic Fluency**	23	-0.06 (-0.37 to 0.23)	13	-0.12 to -0.03

4^th^ column shows the number of patients at the red points in the ‘Patient Atlas’. The last column shows the *S%* scores in the regions of the ‘Functional Atlas’, corresponding to the red points on the ‘Patient Atlas’.

^**a**^*Funct Atlas* = Functional atlas

^b^*Min* = minimum

^c^*Max* = maximum

^**d**^*STN* = subthalamic nucleus

^**e**^*UPDRS* = Unified Parkinson’s Disease Rating Scale Part III

^**f**^*GPm* = medial globus pallidu*s*

### STN DBS group

#### UPDRS III functional and patient atlases

**Fig 1:**
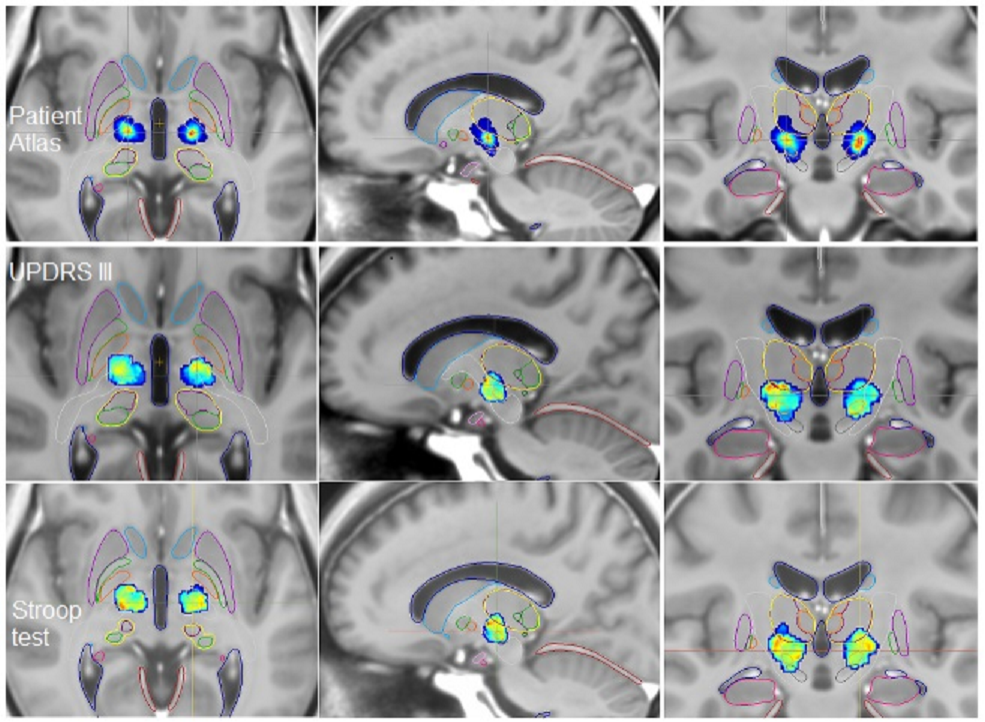
Axial, sagittal and coronal views of the ParkMedAtlis template showing the ‘Patient Atlas’ *(first row)*, the ‘Functional Atlases’ built on UPDRS III analysis *(second row)* and on Stroop analysis *(third row)* in patients with STN DBS at 6 months postoperatively. Amygdala (*light pink*), hippocampus (*pink*), putamen (*violet*), GPm (*orange*), internal capsule (*white*), lateral pallidum (*green*), caudate nucleus (*light blue*), thalami (*yellow*), substantia nigra (*grey*), STN (*light orange*), cerebellar tent (*red*), ventricles (*dark blue*).

We obtained a ‘Patient Atlas’ (Fig 1, *first row*) showing most of the patients stimulated in the supero-lateral part of the two STN. In the region with the maximum of patients on the ‘Patient Atlas’ (red points on Fig 1, *first row*), the UPDRS III *S%* score was represented in green to yellow colors bilaterally on the ‘Functional Atlas’ (Fig 1, *second row*), meaning an *S%* score between 0.17 and 0.3 (Table 3), suggesting an increase in the postoperative score. On the right ‘Functional atlas’, there were high red superior points on coronal view with an *S%* score of 0.35 (Fig 1, *second row*), representing 3 patients on the ‘Patient Atlas’ (Fig 1, *first row*).

#### Stroop functional and patient atlases

We obtained the same ‘Patient Atlas’ (Fig 1, *first row*) as for the UPDRS III analysis. In the region of the red points on the ‘Patient Atlas’, the Stroop *S%* score was represented in green to orange colors bilaterally (Fig 1, *third row*), meaning an *S%* score between -0.02 and 0.06 (Table 3), suggesting a global stability of the postoperative score.

#### Semantic fluency and patient atlases

**Fig 2:**
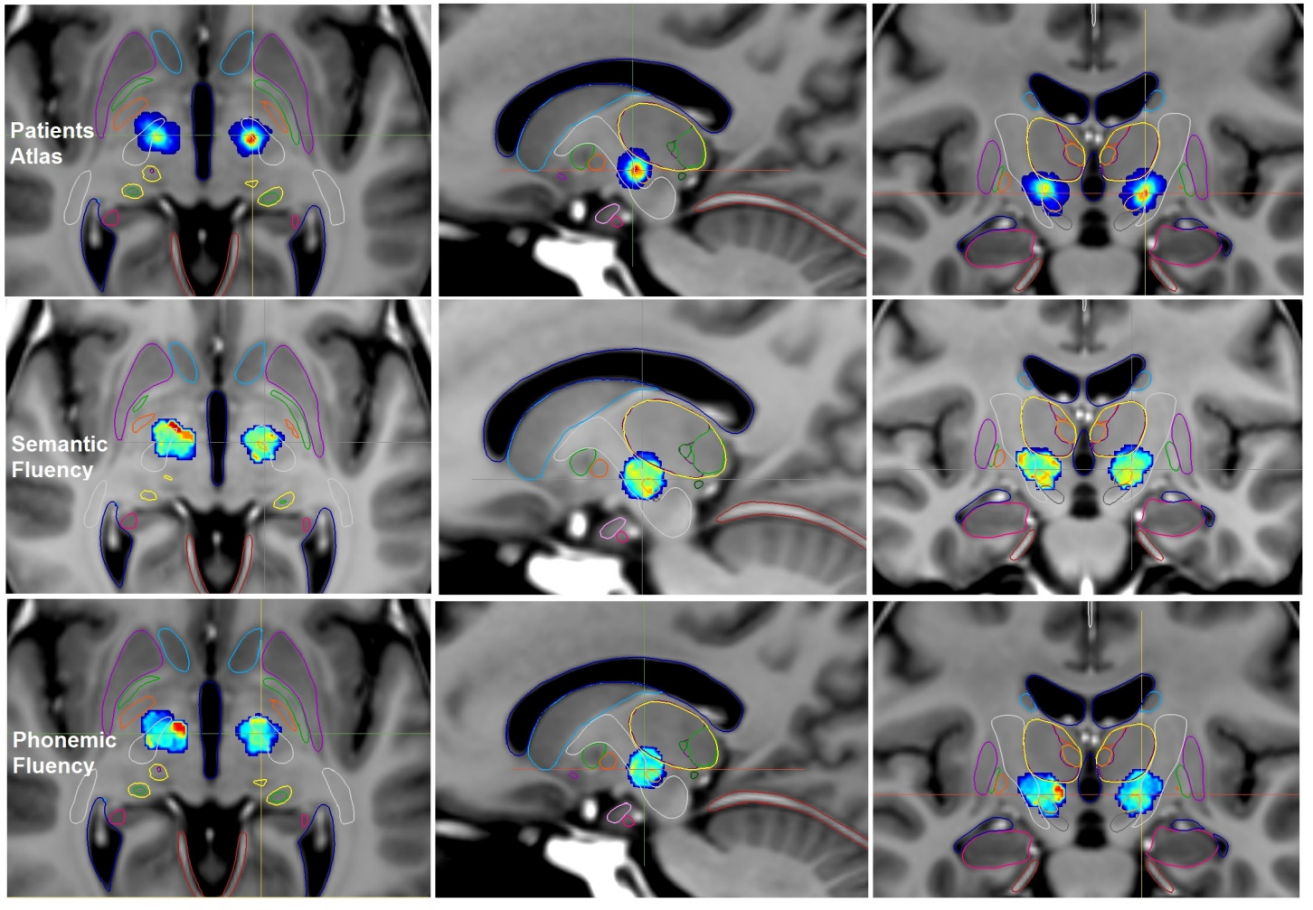
Axial, sagittal and coronal views of the ParkMedAtlis template showing the ‘Patient Atlas’ *(first row)*, the ‘Functional Atlases’ built on verbal fluency analysis *(second row*, semantic fluency and *third row*, phonemic fluency*)* in patients with STN DBS at 6 months postoperatively. Amygdala (*light pink*), hippocampus (*pink*), putamen (*violet*), GPm (*orange*), internal capsule (*white*), lateral pallidum (*green*), caudate nucleus (*light blue*), thalami (*yellow*), substantia nigra (*grey*), STN (*light orange*), cerebellar tent (*red*), ventricles (*dark blue*).

We obtained a ‘Patient Atlas’ (Fig 2, *first row*) showing most of the patients stimulated in the supero-lateral part of the two STN. In the region with the maximum of patients on the ‘Patient Atlas’ (red points on Fig 2, *first row*), the verbal fluency *S%* score was represented in green color bilaterally (Fig 2, *second row*), meaning an *S%* score between -0.17 and -0.05 (Table 3), suggesting a slight decrease in the postoperative score. On the right ‘Functional atlas’, there were orange points around the STN with an *S%* score of 0.13 (Fig 2, *second row*), representing only two patients on the ‘Patient Atlas’.

#### Phonemic fluency and patient atlases

We obtained the same ‘Patient Atlas’ (Fig 2, *first row*) as for the semantic fluency analysis. In the region of the red points on the ‘Patient Atlas’, the phonemic fluency *S%* score was represented in blue-green colors bilaterally (Fig 2, *third row*), meaning an *S%* score between 0.03 and 0.07 (Table 3), suggesting a global stability of the postoperative score. On the right ‘Functional atlas’, there were high red superior and medial points to the STN with an *S%* score of 0.4 (Fig 2, *third row*), representing only one patient on the ‘Patient Atlas’.

### GPm DBS group

#### UPDRS III functional and patient atlases

**Fig 3:**
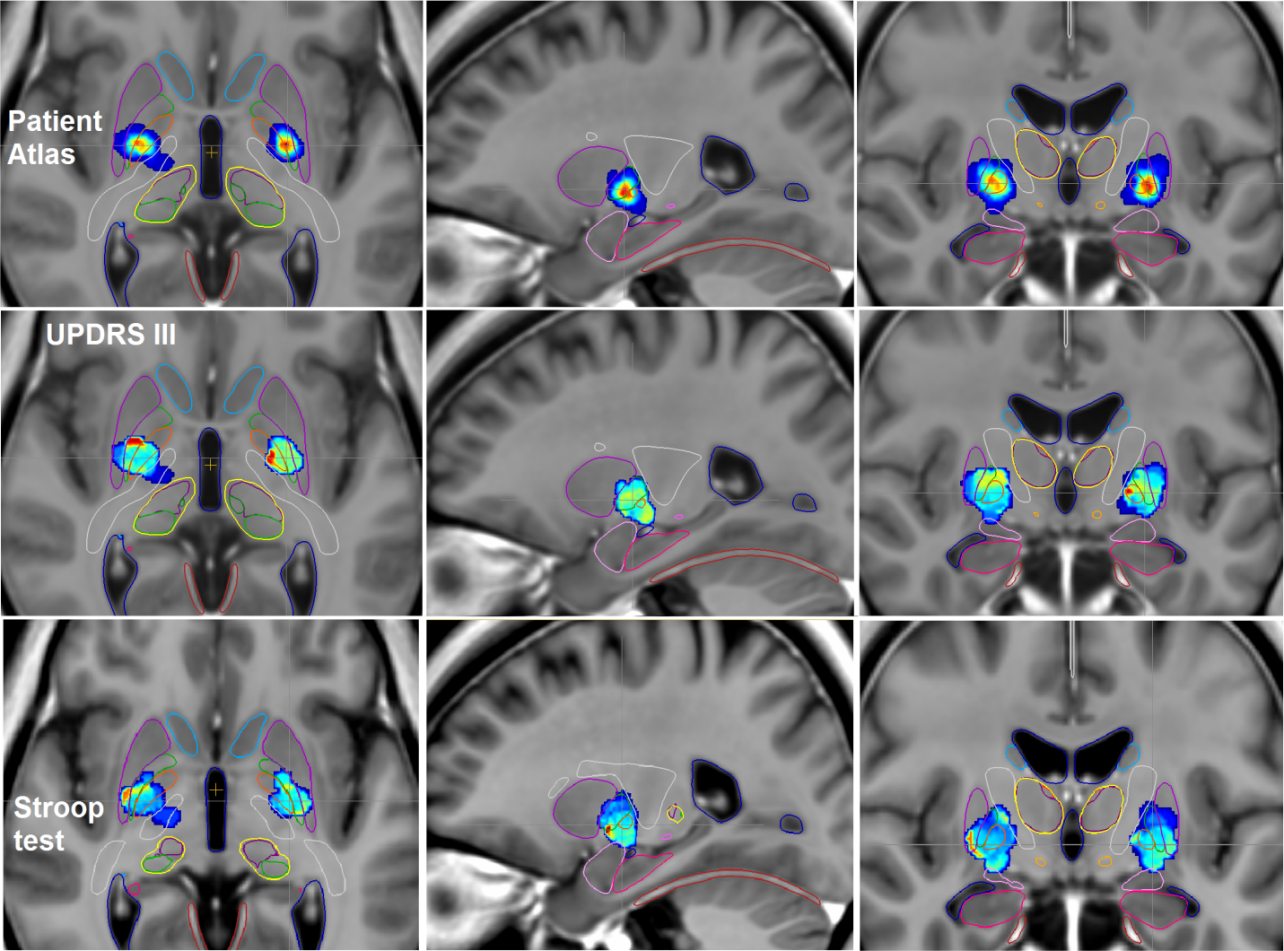
Axial, sagittal and coronal views of the ParkMedAtlis template showing the ‘Patient Atlas’ *(first row)* and the ‘Functional Atlas’ built on UPDRS III analysis *(second row)* and on Stroop analysis *(third row)* in patients with GPm DBS at 6 months postoperatively. Amygdala (*light pink*), hippocampus (*pink*), putamen (*violet*), GPm (*orange*), internal capsule (*white*), lateral pallidum (*green*), caudate nucleus (*light blue*), thalami (*yellow*), substantia nigra (*grey*), STN (*light orange*), cerebellar tent (*red*), ventricles (*dark blue*).

We obtained a ‘Patient Atlas’ (Fig 3, *first row*) showing most of the patients stimulated in the postero-ventral part of the two GPm, more laterally on the left than on the right side. In the region with the maximum of patients on the ‘Patient Atlas’ (red points on Fig 3, *first row*), the UPDRS III *S%* score was represented in green colors bilaterally on the ‘Functional Atlas’ (Fig 3, *second row*), meaning an *S%* score between 0.12 and 0.26 (Table 3), suggesting an increase of the postoperative score. On the ‘Functional atlas’, there were red points located in the right lateral globus pallidus with an *S%* score of 0.54, representing only one patient on the ‘Patient Atlas’, and red points medially in the left GPm with an *S%* score of 0.54, representing only one patient on the ‘Patient Atlas’ (Fig 3, *second row*).

#### Stroop functional and patient atlases

We obtained the same ‘Patient Atlas’ (Fig 3, *first row*) as for the UPDRS III analysis. In the region of the red points on the ‘Patient Atlas’ (Fig 3, *first row*), the Stroop *S%* score was represented in blue colors bilaterally on the ‘Functional Atlas’ (Fig 3, *third row*), meaning an *S%* score between 0.005 and 0.03 (Table 3), suggesting a global stability of the postoperative score. On the right Stroop atlas, there were red points located in the ventral putamen with an *S%* score of 0.35 (Fig 3, *third row*), representing only two patients on the ‘Patient Atlas’.

#### Semantic fluency and patient atlases

**Fig 4:**
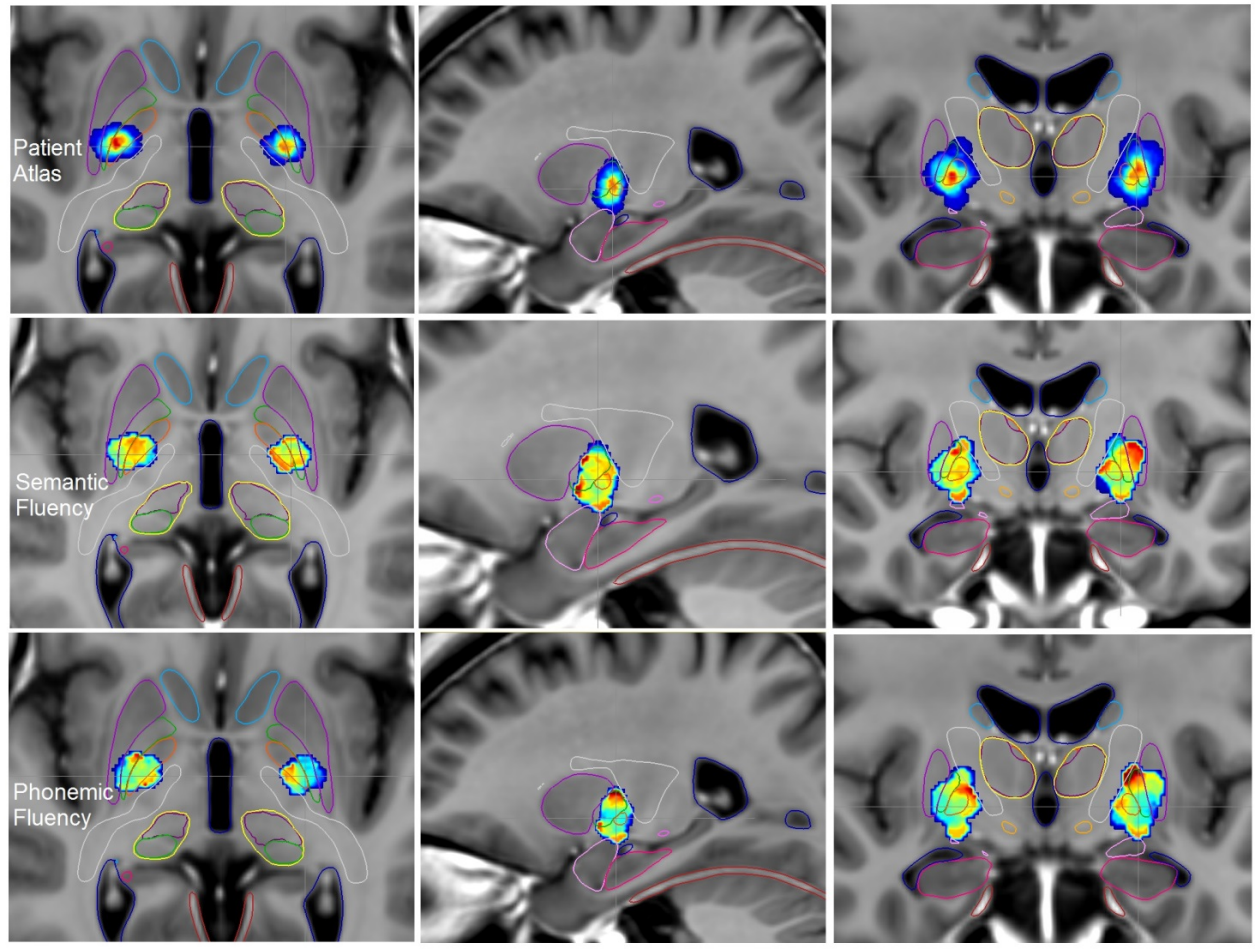
Axial, sagittal and coronal views of the ParkMedAtlis template showing the ‘Patients Atlas’ *(first row)*, the ‘Functional Atlas’ built on verbal fluency analysis *(second row*, semantic fluency and *third row*, phonemic fluency*)* in patients with GPm DBS at 6 months postoperatively. Amygdala (*light pink*), hippocampus (*pink*), putamen (*violet*), GPm (*orange*), internal capsule (*white*), lateral pallidum (*green*), caudate nucleus (*light blue*), thalami (*yellow*), substantia nigra (*grey*), STN (*light orange*), cerebellar tent (*red*), ventricles (*dark blue*).

We obtained a ‘Patient Atlas’ (Fig 4, *first row*) showing most of the patients stimulated in the postero-ventral part of the two GPm, more laterally on the left than on the right side. In the region with the maximum of patients on the ‘Patient Atlas’ (red points on Fig 4, *first row*), the semantic fluency *S%* score was represented in yellow to orange colors bilaterally on the ‘Functional Atlas’ (Fig 4, *second row*), meaning an *S%* score between -0.02 and 0.06 (Table 3), suggesting a global stability of the postoperative score.

#### Phonemic fluency and patient atlases

We obtained the same ‘Patient Atlas’ (Fig 4, *first row*) as for the semantic fluency analysis. In the region of the red points on the ‘Patient Atlas’ (Fig 4, *first row*), the phonemic fluency *S%* score was represented in blue-green bilaterally on the ‘Functional Atlas’ (Fig 4, *third row*), meaning an *S%* score between -0.12 and -0.03 (Table 3), suggesting a global stability of the postoperative score, perhaps a slight decrease. On the left ‘Functional atlas’, there were red points inside and up the lateral globus pallidus with an *S%* score of 0.23 (Fig 4, *third row*), representing only two patients on the ‘Patient Atlas’.

## Discussion

The present article is the first, to our knowledge, to analyse the motor and neuropsychological effects of the GPm and STN DBS with functional atlases. These were built for a group analysis, at 6 months postoperatively showing in 3D and in colour the motor and neuropsychological outcome. The *S%* scores used for building the atlases were not analysed with statistical tests. Only a visual and numerical analysis of the atlases is currently feasible. We did not want to compare the atlases between the two groups. Table 1 presented the differences between the motor and neuropsychological scores in the two groups.

Concerning the results after STN DBS, the patients showed significant motor improvement, also visible on the UPDRS III Functional Atlas (Fig 1). In a previous study including our first 30 patients with STN DBS, we demonstrated, with the help of anatomo-clinical atlases, a discrepancy between good motor improvement in the supero-lateral part of the STN and impairment of the fluencies in the same region [16]. Nevertheless, these first anatomo-clinical atlases did not take into account the volume of tissue activated (VTA) nor the fusion of the atlases on the ParMedAtlis template to visualize the STN and the adjacent structures accurately. The impairment of verbal fluency is the most commonly reported side-effect after STN DBS [6–8,17,25,35]. In our series, patients after STN DBS did not present a significant decline in semantic and phonemic fluency, while the semantic fluency functional atlas and its median *S%* score suggested a slight deterioration (Fig 2). The cause of postoperative fluency changes after STN DBS are still debated both in term of cognitive and cerebral network mechanisms [17,25,35,36]. Houvenaghel et al. [25] compared metabolic and executive changes between patients with and without postoperative semantic or phonemic deterioration following STN-DBS. They did not find any group differences either in frontal area, or in executive changes but observed group differences in response speed and apathy. Okun et al [35] and Mikos et al. [17] have shown after unilateral STN DBS (equally left and right) a decreased phonemic fluency correlated with the VTA on ventral contacts of the STN. At dorsal or optimal contacts inside the STN, they did not observe any phonemic fluency decline seven months postoperatively. Our patients were stimulated bilaterally and studied only at their optimal contacts, i.e. at the supero-lateral part of the STN. The mean coordinates of the STN target were about 14 mm laterally, 16 mm posterior to the AC and almost one mm under the AC-PC line, in agreement with the 3D representation on the ‘Patient Atlases’ (Figs 1 and 2). This target was more lateral than those previously reported [4,37], meaning perhaps that we avoid the more ventral zone, at risk of verbal fluency deterioration. We explain this result by the fact that the neurosurgeon carried out direct targeting of the STN on 3T T2 MRI instead of probabilistic targeting based on the Talairach atlas [38].

Now the ‘Functional Atlases’ provide us with the effects of GPm stimulation, also concerning the lateral globus pallidus and the adjacent structures. We obtained similar results to those previously published in the literature [10,35,39] about motor improvement after GPm DBS. We observed motor improvement by stimulating the postero-ventral part of the GPm. According to the Talairach atlas [38], the mean coordinates of the GPm target were about 24.7 mm laterally, 11.8 mm posterior to the AC and on the AC-PC line, in agreement with the 3D representation on the ‘Patient Atlases’ (Figs 3 and 4). According to Laitinen et al. [26,40] and Schaltenbrand atlas [41], the GPm targeting was described as 2 to 3 mm anterior to the mid-commissural point between AC and PC, 18 to 2mm lateral and 6 to 7 mm below the intercommissural line. In our study, the average contact was located more laterally and at a shallower depth, perhaps due to a large third ventricle because of severe cortico-subcortical atrophy induced by the PD.

After GPm DBS we observed at 6 months stability in the Stroop test and semantic fluency, and only a reduction in phonemic fluency in the postero-ventral of the GPm on the “Functional Atlas”, whereas behavioral statistical tests did not highlight any change in neuropsychological tests. This point is important in patients with preoperative cognitive impairment. After the Mikos et al. [17] study in STN DBS, Dietz et al. [42] similarly studied the link between the VTA and decline in verbal fluency in 14 patients with unilateral GPm DBS. There was no significant relationship between the location of the VTA within the GPm and verbal fluency performance.

Perhaps the GPm stimulation has no neuropsychological and psychiatric effects thanks to its well-separated connections to the motor and limbic circuits of the basal ganglia, and also to its quasi-absence of direct cortical connections, unlike the STN. Pallidotomy produced marked motor improvement in PD patients without their experiencing cognitive deterioration according to the lesion location [43]. More anterior lesions affecting the limbic part of the GPm led to postoperative cognitive deterioration [43], not seen in a meta-analysis of patient outcome after GPm stimulation [11,44]. Inside the motor parts of the lateral globus pallidus and GPm, Yelnik et al. [45] described different effects of the stimulation on the motor symptoms. They observed that rigidity was improved whatever the site of stimulation in the motor globus pallidus whereas akinesia was improved only by stimulating the lateral globus pallidus. The site where akinesia was most pronounced in the motor GPm was also the site which improved dopamine-induced dyskinesia. The contacts which stimulated the lateral globus pallidus were dorsal on the electrode and the contacts stimulating the GPm were ventral. In our study, most patients were stimulated at the junction between the GPm and the lateral globus pallidus, especially visible on the left side of the ‘Patient Atlas’ (Fig 3). The GPm being larger than the STN, GPm stimulation sometimes needs to stimulate two contacts thus potentially mixing motor improvements on akinesia, rigidity and dopamine-induced dyskinesia.

We built the anatomo-clinical 3D Atlases within a common spatial referential, the ParkMedAtlis template, by automatically detecting the contacts of the electrodes on the postoperative CT and by using a validated registration tool to obtain a reliable localization of the clinical and anatomical data on the Parkinson template. Some limits to our method were 1) the use of different registration steps to obtain volume of stimulation on the ParkMedAtlis template, 2) the estimation of the VTA by a 3mm Gaussian around the stimulated contacts and 3) the arbitrary color coding of the *S%* score, 4) the lack of a connectivity approach. Concerning the registration tools, we have already published the comparison between three registration tools and the validation of each tool in terms of registration errors [16,29]. The registration approach used in this paper was the most accurate, combined with the use of a multi-subject rather than a mono-subject template [16]. Our choice for modeling the neural response to stimulation was based on the current state of reflection carried out by Butson et al. [34]. The authors, regarding the long time-consuming processing of their stimulation model, proposed an online tool easier to use in our case [34]. Further studies are needed on the modeling of a clinical score by color scale to render it more informative for clinicians. A recent approach to creating functional atlases has been to investigate not only the anatomical and functional target in STN DBS, but also the connectivity between the STN and cortical areas [46,47]. Although we find similar therapeutic areas in the STN to those found by Akram et al. [46] (i.e. the supero-lateral part of the STN), fiber tractography was not available at the time of our study and prevented us adopting a connectomic approach. Our goal was to use the ‘Functional and Patient Atlases’ in clinical practice of DBS, without needing engineer time. Nevertheless, the functional atlases today remain a 3D tool more easily readable on a computer than on 2D. We developed these functional atlases into a specific DBS software package (PyDBS) used in a clinical practice to plan the electrodes’ trajectories [30]. Briefly, the first step is composed of six processing modules to obtain the brain-ventricle and basal-ganglia segmentations projections from atlas to patient space. During the first step the neurosurgeon (CH) defined the AC and PC points on the preoperative MRI. The second step is the definition of the target inside the chosen nucleus and the entry point. To choose the target, the neurosurgeon defined the nucleus target with the help of the anatomical information of the T2 sequence, then verified the target point according to the coordinates from the AC point and finally, used the motor functional atlas to verify the target point. All these data are recording inside the PyDBS software. A further study should analyze how the functional atlases could be a help in targeting a nucleus, either the STN or the GPm according to the clinical scores of a new patient. A further possibility is to use the atlases for postoperative programming to find the best contacts to produce a good motor effect with fewer neuropsychological side effects.

We proposed new functional atlases to produce a 3D representation of DBS motor and neuropsychological effects. The Functional Atlases were larger than the targeted nucleus, because of the VTA estimated by a 3mm Gaussian around the stimulated contacts, and because of the anatomical variability of the PD patients, which was registered on the ParkMedAtlis template. We also defined larger atlases for the GPm stimulation than for the STN because of the higher average effective voltage necessary to obtain a clinical effect for GPm stimulation [48]. Functional Atlases were asymmetric regardless of the score. For motor results, this was more understandable because of the frequent asymmetry of the PD. For neuropsychological results, one explanation could be the asymmetry of the stimulated contacts in one subject with bilateral DBS, which is why we were interested in the region with the maximum of patients on each Patient Atlas. We suggest that this region may be the more homogeneous on the Functional Atlas inside a stimulated zone. Outside this region, the Functional Atlas represented a minority of the patients. One future improvement of the Functional Atlas could be a multi-functional atlas representing several *S%* scores. One other idea is to validate the Functional Atlas with the help of validation methods using a leave-one-out procedure. Using this kind of method, we will test the performance of the Functional Atlas to predict the patients’ outcome.

## Conclusions

We developed new Functional anatomo-clinical Atlases to visualize the motor and neuropsychological consequences at 6 months of STN and GPm stimulation in patients with PD. These Atlases provided us with confirmation of the motor improvement in the supero-lateral part of the STN.We also proposed an effective, more lateral targeting of the GPm in PD because of the cortico-subcortical atrophy induced by the disease. Our goal is to use these ‘Functional Atlases’ prospectively in further patients to improve their targeting, thus ensuring a shorter planning step on the day of the surgery as well as better outcomes from the motor and neuropsychological points of view.

## Supporting information

S1 File Data protection certificate. (PDF)

S2 File Data set. (XLS)
