# Correction: Functional atlases for analysis of motor and neuropsychological outcomes after medial globus pallidus and subthalamic stimulation

**DOI:** 10.1371/journal.pone.0223693

**Published:** 2019-10-03

**Authors:** The PLOS ONE Staff

## Notice of republication

An incorrect version of S2 File was published in error. The publisher apologizes for this error. This article was republished on September 25, 2019 to correct for this error. Please download this article again to view the correct version.
