# Supplementary material for: Functional atlases for analysis of motor and neuropsychological outcomes after medial globus pallidus and subthalamic stimulation
Source: PLoS One. 2018 Jul 13;13(7):e0200262. doi: 10.1371/journal.pone.0200262 (PMC6044526; doi:10.1371/journal.pone.0200262)
Supplement: S1 File — (PDF) [file pone.0200262.s001.pdf]

# Inter Deposit Digital Number

Certificat délivré par

**Agence pour la Protection des Programmes**

54 rue de Paradis - 75010 PARIS - FRANCE / T. +33(0)1 40 35 03 03 / F. +33(0)1 40 38 96 43

## IDDN.FR.001.110034.000.S.P.2017.000.31230

(1) (2) (3) (4) (5) (6) (7) (8) (9) (10)

Pour l'œuvre : **PyDBS-DATA version 01 en date du 12 janvier 2017**

Identité des titulaires de droits* :

**UNIVERSITÉ DE RENNES 1**
**2 rue du Thabor**
**CS 46510**
**35065 RENNES CEDEX**
**FRANCE**
**Siren : 193509361**

**INSERM**
**101 rue de Tolbiac**
**75654 PARIS CEDEX 13**
**FRANCE**
**Siren : 180036048**

**CHU DE RENNES**
**2 rue Henri Le Guilloux**
**35033 RENNES CEDEX 9**
**FRANCE**
**Siren : 263500076**

Adhérent sous le numéro : **92.35.2610**
Support utilisé : 1 CD en double exemplaire

Logibox conservée par l'adhérent : 114980
Logibox conservée par l'APP : 114981

Fait à Paris, le 09/03/2017

Le Président

AGENCE POUR LA PROTECTION DES PROGRAMMES
T. +33 (0)1 40 35 03 03 – F. +33 (0)1 40 38 96 43
54 rue [illegible] 85 385 844 – APE 94997
APP.ASSO.FR

(1) Inter Deposit Digital Number
(2) Nationalité de l'œuvre
(3) Numéro de l'organisme d'enregistrement
(4) Numéro d'ordre de l'enregistrement
(5) Numéro de version
(6) Type d'enregistrement
(7) Type de l'œuvre
(8) Année d'enregistrement
(9) Zone réservée (Clé intégrité)
(10) Classe du produit

** Le titulaire s'engage à informer l'APP de toute cession ou aliénation,totale ou partielle, de ses droits de propriété intellectuelle.*
*Seules les inscriptions de type S et C permettent un éventuel accès au programme source.*
